# Alterations in interhemispheric functional and anatomical connectivity are associated with tobacco smoking in humans

**DOI:** 10.3389/fnhum.2015.00116

**Published:** 2015-03-09

**Authors:** Humsini Viswanath, Kenia M. Velasquez, Daisy Gemma Yan Thompson-Lake, Ricky Savjani, Asasia Q. Carter, David Eagleman, Philip R. Baldwin, Richard De La Garza, II, Ramiro Salas

**Affiliations:** ^1^Menninger Department of Psychiatry and Behavioral Sciences, Baylor College of MedicineHouston, TX, USA; ^2^Department of Neuroscience, Baylor College of MedicineHouston, TX, USA

**Keywords:** resting state functional connectivity, diffusion tensor imaging, tobacco smoking, interhemispheric connectivity, corpus callosum

## Abstract

Abnormal interhemispheric functional connectivity correlates with several neurologic and psychiatric conditions, including depression, obsessive-compulsive disorder, schizophrenia, and stroke. Abnormal interhemispheric functional connectivity also correlates with abuse of cannabis and cocaine. In the current report, we evaluated whether tobacco abuse (i.e., cigarette smoking) is associated with altered interhemispheric connectivity. To that end, we examined resting state functional connectivity (RSFC) using magnetic resonance imaging (MRI) in short term tobacco deprived and smoking as usual tobacco smokers, and in non-smoker controls. Additionally, we compared diffusion tensor imaging (DTI) in the same subjects to study differences in white matter. The data reveal a significant increase in interhemispheric functional connectivity in sated tobacco smokers when compared to controls. This difference was larger in frontal regions, and was positively correlated with the average number of cigarettes smoked per day. In addition, we found a negative correlation between the number of DTI streamlines in the genual corpus callosum and the number of cigarettes smoked per day. Taken together, our results implicate changes in interhemispheric functional and anatomical connectivity in current cigarette smokers.

## Introduction

Tobacco abuse is the leading cause of preventable illness and death in developed countries (Peto et al., 1996; Benowitz, 2008). Several studies point to nicotine as the primary addictive component in tobacco (Mansvelder and McGehee, 2002; Benowitz, 2009). The effects of tobacco on brain functioning have been extensively studied. Despite availability of several FDA-approved medications, many (50–75%) of those who attempt to quit fail within the first week (Garvey et al., 1992; Hughes et al., 2004; Baldwin et al., 2011). Thus, a better understanding of the neural effects of tobacco in the human brain is necessary to develop better anti-tobacco therapies.

Both anatomical and functional brain connectivity have been shown to be altered by tobacco smoke. Resting state functional connectivity (RSFC) is a non-invasive brain imaging method that identifies low frequency signal correlations among different areas of the brain (Biswal et al., 2010) and has been repeatedly used to study brain connectivity in tobacco smokers. For example, a study using RSFC in tobacco smokers showed that nicotine (7 mg transdermal nicotine patch for 90 min prior to the scan), improved local and regional network efficiency in limbic and paralimbic regions (Wylie et al., 2012). Under similar conditions, another study found acute nicotine decreased activity in the default mode network and increased activity in extra-striate regions, which could help explain some of the positive effects of nicotine on attention (Tanabe et al., 2011). In addition, prefrontal and limbic networks have shown enhanced connectivity in smokers (Janes et al., 2012), anterior insula connectivity is positively correlated with cue-induced nicotine craving (Moran-Santa Maria et al., 2015), and weaker inter-network connectivity between the default and salience networks was associated to abstinence-elicited nicotine cravings (Lerman et al., 2014; Zorlu et al., 2014). Finally, we have shown that in a highly co-morbid sample of psychiatric patients, the presence of substance abuse is associated with both increased interhemispheric insular and inferior frontal cortical RSFC and decreased FA in several brain areas including the corpus callosum (Viswanath et al., 2015).

Diffusion tensor imaging (DTI) is also a non-invasive brain imaging method that provides information on white matter, by studying the preferential directions of water diffusion within brain areas. Recently, the white matter in the anterior corpus callosum was shown to have lower fractional anisotropy (FA) in smokers (Lin et al., 2013; Savjani et al., 2014) and chronic smokers were shown to exhibit increased fronto-parietal FA in the longitudinal fasciculus (Liao et al., 2011). Also, nicotine patches (21 mg) were shown to acutely increase genual FA (Kochunov et al., 2013). DTI has also been used to study drugs of addiction other than nicotine. For example, widespread changes in DTI parameters were found in abstinent alcoholics (Zorlu et al., 2014), chronic cocaine abuse has been linked to white matter damage (Narayana et al., 2014), and decreased FA was found in several brain areas in heroin dependent subjects (Li et al., 2013).

One characteristic that can be studied using RSFC is interhemispheric functional connectivity. Right/left brain asymmetry is a very important feature of brain architecture (Turk et al., 2003), in fact, each hemisphere is responsible for different mental functions (Gazzaniga, 1967). Accordingly, slight abnormalities in right/left brain connectivity are associated with several psychiatric and neurologic conditions, such as depression, obsessive-compulsive disorder, schizophrenia, autism, and stroke (Pettigrew and Miller, 1998; Spencer et al., 2003; Anderson et al., 2011; Chen and Schlaug, 2013; Guo et al., 2013a,b; Volpato et al., 2013; Wang et al., 2013). Of interest, cocaine use has been shown to decrease interhemispheric RSFC (Kelly et al., 2011), and cannabis use has been shown to both increase and decrease interhemispheric RSFC between specific brain regions (Orr et al., 2013). In terms of the possible involvement of interhemispheric connectivity in drug abuse, it must be noted that the main purpose of the corpus callosum is to transfer information across the midline. Therefore, the above mentioned papers showing callosal alterations in drug abuse point to the possibility of interhemispherical alterations in functional connectivity.

To expand our understanding of how cigarette smokers differ from matched controls, we performed both interhemispheric RSFC (in both sated and deprived conditions) and callosal DTI in tobacco smokers and compared the outcomes to non-smoking, matched controls. We started using whole half-brains, then subdivided the brain into 3 gross regions (frontal, center, caudal) and finally refined the frontal region (where differences were observed) into anatomically defined areas. This strategy allowed us to avoid severe multiple comparisons issues. We hypothesize interhemispheric RSFC and FA will differ between cigarette smokers and matched non-smoking controls.

## Methods

### Subjects

Data were collected from 32 smokers (19 male; 12 African American, 7 Hispanic, 13 Caucasian, age = 42(2) years; mean(sem)). Subjects were screened for use of illicit substances and evidence of psychiatric disorders. Subjects who tested positive on a urine toxicology analysis for any illicit substance or coded for any Axis I disorder according to the Mini-International Neuropsychiatric Interview, were excluded. Dependence of nicotine was assessed using Fagerström Test for Nicotine Dependence (FTND; Heatherton et al., 1991). All smokers were scanned after being tobacco-deprived for 24 h. Of those, 17 were also scanned under “sated” conditions (these were smoking as usual, and were asked whether they would like to smoke right before being scanned; 11 male; 8 African American, 3 Hispanic, 7 Caucasian; age = 44(2) years). Tobacco deprivation was verified by exhaled carbon monoxide (CO). The average CO for the deprived group was 3.2(0.3) parts per million (ppm); the average CO for the sated group was 16.0(1.2). In addition, 28 non-smoker controls were scanned (11 male; 9 African American, 13 Caucasian, 6 Hispanic; age = 36(3) years). Subjects were scanned on a Siemens 3T Trio Magnetom scanner at Baylor College of Medicine Center for Advanced Magnetic Resonance Imaging (CAMRI). All procedures were approved by Baylor College of Medicine Internal Review Board. All participants read and signed an Informed Consent to participate in this research protocol.

### Structural Imaging

The structural scan was an MPRAGE sequence TE = 2.66 ms, TR = 1200 ms, flip angle = 12°, 256 × 256 matrix, 160 1 mm axial slices (1 × 1 × 1 mm voxel). Structural images were used to rule out possible structural defects (tumors, etc.), and to co-register with functional data.

### Resting State Imaging

Subjects were scanned while resting for 5 min. An “X” was displayed in the screen. No specific instruction was given except to “let your mind wander”. Participants were not told to fixate on the “X”, but it was there if they wanted an image to focus on. Scanner parameters: 5 min resting state scan (gradient EPI sequence), whole brain, 3 × 3 × 3 mm voxels, TE = 40 ms, TR = 2 s, flip angle = 90°.

### PreProcessing

Single subjects’ functional image time-series were realigned to the first image of the series. The mean realigned functional image was then coregistered to the structural scan and normalized to the standard Montreal Neurological Institute (MNI) atlas EPI space. Next, images were smoothed with a full width at half-maximum (FWHM) Gaussian Smoothing kernel of 6 mm. The structural scan was normalized to MNI space. SPM8 was used for image pre-processing and statistical analysis (Ashburner and Friston, 1999).

Subjects who moved 2 mm or more (translation) during the 5 min of resting state were not included in the RSFC analysis. Eight subjects were excluded from the deprivation condition, 4 from the sated condition, and 4 from the control group. Regions of interest (ROIs) for hemispheres and hemisphere-thirds (frontal, medial, caudal) cortex were created in AFNI using the included MNI atlas. CONN, a Matlab toolbox (Whitfield-Gabrieli and Nieto-Castanon, 2012), was used in the RSFC analysis. All default parameters in CONN were used in the analysis. CONN processing includes a gray matter segmentation step; therefore no segmentation was performed during preprocessing. The movement files from preprocessing, the CSF, and white matter signals were used as regressors of no interest. The data was filtered in CONN with a frequency range of [0.008, 0.09] Hz. After processing the data through CONN, the Fisher-transformed correlation coefficients between the different seeds for each subject were identified. In order to make sure the effects we observed were group-specific, we calculated the total brain volume for each subject by adding up gray and white matter to see if there were volume differences between groups.

### DTI Imaging

Scanner acquisition parameters for DTI were: voxel size: 2 × 2 × 2 mm, slices: 61 transversal slices (2 mm thick with no gap), phase encoding direction: A-P, FOV: 256 × 256 mm, TR: 9.4 s, TE: 91 ms, matrix size: 128 × 128, echo spacing: 73 ms, diffusion directions: 71 unique directions at b_0_ = 1000 s/mm^2^ with 8 repetitions at b_0_ = 0 s/mm^2^, duration: 12:32 min. Due to FOV and slice selection, the cerebellum and posterior occipital lobes were not fully captured for every subject. After acquiring the images, the Diffusion Toolkit software was used to reconstruct the diffusion MR images with an angle threshold of 45° and an inverted Z orientation patch. Finally, the Trackvis software (A.A. Martinos Center for Biomedical Imaging) was used to display and analyze the data. We focused on 2 ROI to analyze the tractography. The first ROI (radius = 2 mm) was in the genu of the corpus callosum. The second ROI (radius = 2 mm) was in the splenium of the corpus callosum. ROIs were manually placed by an experimenter blind to smoking status. The genual ROI was placed in the frontal segment and above the curve of the corpus callosum. The second ROI was placed at the center of the splenium. See Figure 4 for a depiction of these ROIs. Through each ROI we analyzed number of streamlines and mean FA. DTI data was obtained during the deprived session.

**Figure 1:**
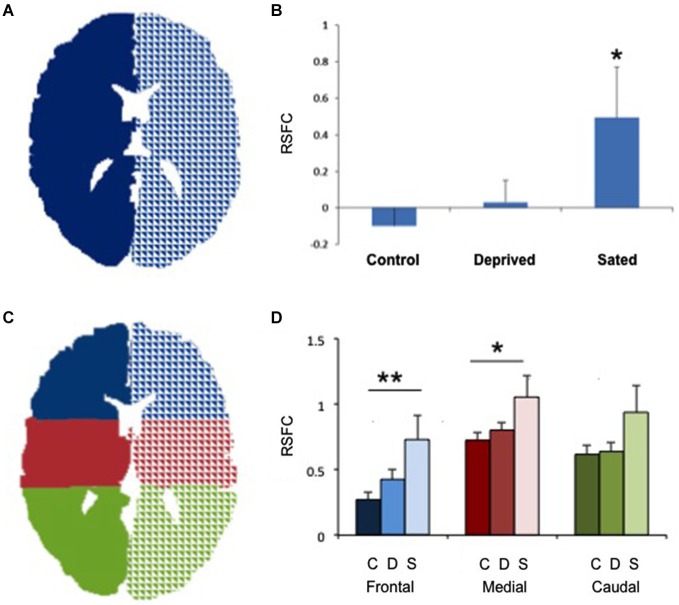
**Increased interhemispheric connectivity in sated smokers. (A)** The two seeds used to study right/left connectivity. Only one central section is shown. **(B)** Interhemispheric resting state functional connectivity (RSFC) in non-smokers, deprived smokers and sated smokers. *p < 0.02 vs. controls. **(C)** The 6 seeds used for frontal, medial and caudal interhemispheric connectivity assessment. Only one central section is shown. **(D)** Interhemispheric resting state functional connectivity (RSFC) in non-smokers (C, darker bars), deprived smokers (A, medium bars) and sated smokers (S, lighter bars) in frontal, medial, and caudal sections. **p < 0.005, *p < 0.05 vs. controls in the same area.

**Figure 2:**
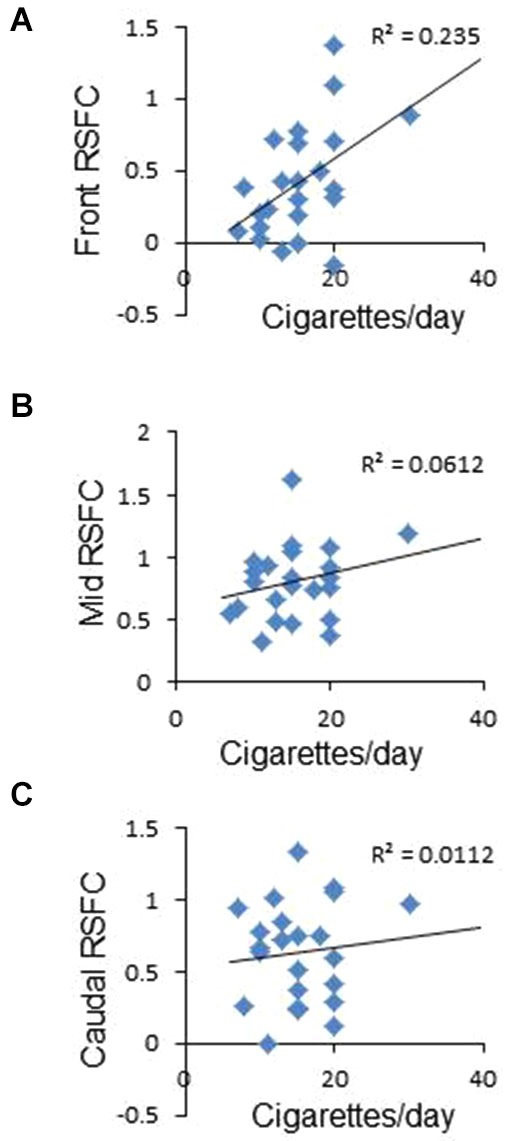
**Correlation between frontal interhemispheric functional connectivity in deprived subjects and average number of cigarettes smoked per day. (A)** Positive significant correlation between frontal RSFC and cigarettes/day. **(B,C)** No correlation between medial **(B)** or caudal **(C)** RSFC and cigarettes/day.

**Figure 3:**
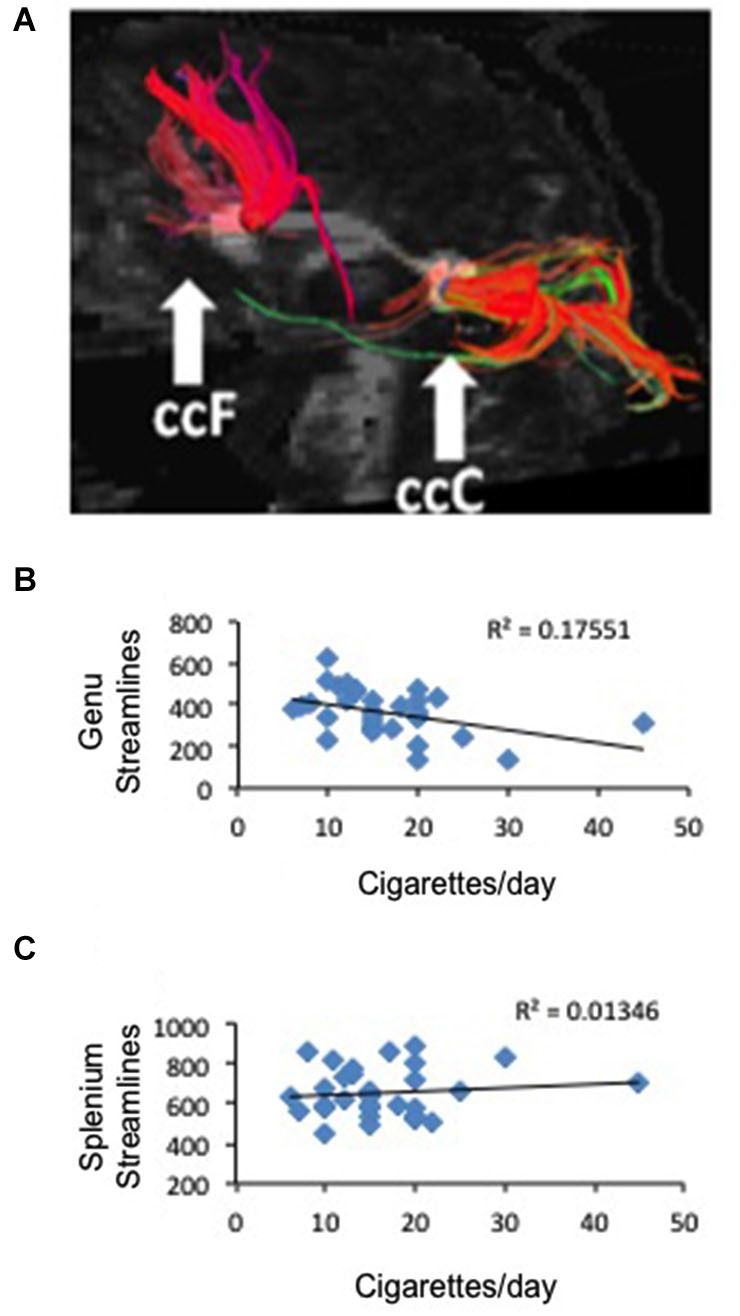
**Correlation between frontal anatomical connectivity as measured with DTI and average number of cigarettes smoked per day. (A)** Two callosal ROIs (genu and splenium) and streamlines that pass through each ROI (one representative subject is shown) **(B)** Significant negative correlation between number of streamlines in the genual ROI and average number of cigarettes per day *p* < 0.02. **(C)** No correlation between number of streamlines in the splenium ROI and average number of cigarettes per day.

**Figure 4:**
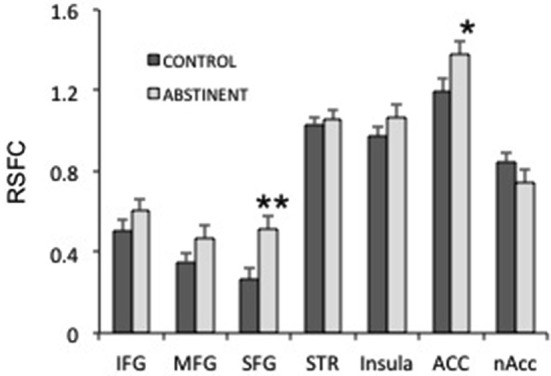
**Interhemispheric resting state functional connectivity (RSFC) in several frontal regions**. IFG, MFG, SFG: inferior, medial and superior frontal gyri; STR: striatum; ACC: anterior cingulate cortex; nAcc: nucleus accumbens. **p_uncorrected_ < 0.005; *p_uncorrected_ < 0.05.

### Statistics

Sudent’s *t*-tests were used to compare smokers in sated and abstinent conditions (paired *t*-test), sated smokers and non-smokers, and abstinent smokers and non-smokers (unpaired *t*-tests). ANOVA was used to compare RSFC and FA/streamlines in the 3 regions studied (frontal, central, caudal). Linear regressions were performed by correlating the number of cigarettes per day or the Fagerstrom Test for Nicotine Dependence (Heatherton et al., 1991) scores to imaging parameters (RSFC between two seed regions or number of DTI streamlines in a region of interest). All data are expressed as mean ± sem. Significance was set at *p* < 0.05 for both linear regression and ANOVA. To compare interhemispheric RSFC between specific frontal regions of the brain, we used student’s *t* test and Bonferroni correction for multiple comparisons.

## Results

No statistically significant group differences were found for sex, age or total brain size. Sated smokers showed a statistically significant increase in interhemispheric connectivity as compared to non-smokers (Figures 1A,B, *p* < 0.05). In contrast, there was no difference in interhemispheric connectivity in non-smokers vs. deprived smokers (Figure 1, *p* = 0.4, Nabs = 24, Ncont = 23), though the difference between sated and deprived smokers approached significance (Figures 1A,B, *p* = 0.08, Nabs = 24, Nsat = 13). We then divided the brain in 3 regions: frontal, medial and caudal (Figure 1C), and studied the interhemispheric RSFC between right and left for each of the regions. We found significant differences in interhemispheric RSFC between sated smokers and controls in the frontal region (*F*_(2,57)_ = 5.05, *p* < 0.005) and medial region (*F*_(2,57)_ = 3.32, *p* < 0.05), but there was not a statistically significant difference in the caudal region (*F*_(2,57)_ = 2.38, *p* > 0.05; Figure 1D). To verify whether the enhanced interhemispheric connectivity observed was a result of increased general connectivity in the sated state, we studied the RSFC between right frontal and right caudal ROIs, and left frontal and left caudal ROIs. No significant difference was observed between sated smokers, deprived smokers, and controls when same-side fronto/caudal RSFC was studied.

To study whether frontal interhemispheric RSFC correlates with meaningful parameters related to tobacco smoking, we performed correlations between deprived or sated frontal interhemispheric RSFC and the average number of cigarettes smoked per day. As shown in Figure 2A, there was a positive correlation between deprived frontal interhemispheric RSFC and the number of cigarettes per day (*r*^2^ = 0.235, *p* < 0.02). No significant association was detected for the sated condition (*p* > 0.05) or in the medial and caudal regions (Figures 2B,C).

To study the white matter that connects the right and left brain, we defined two ROIs in the genu and splenium of the corpus callosum and used tractography to measure the number of streamlines that traverse each ROI in smokers and non-smoking controls and the FA associated to those tracts (Figure 3A). We did not find a difference between deprived smokers and non-smokers in number of streamlines in either genu (*F*_(1,62)_ = 0.006, *P* = 0.9) or splenium (*F*_(1,62)_ = 2.56, *P* = 0.11). However, FA values were significantly smaller in smokers than in controls, in both genu (FA_control_ = 0.551 ± 0.005, FA_smoker_ = 0.507 ± 0.009; *F*_(1,61)_ = 19.7, *P* < 0.0005) and splenium (FA_control_ = 0.617 ± 0.005, FA_smoker_ = 0.582 ± 0.007; *F*_(1,61)_ = 15.6, *P* < 0.0005). Based on the RSFC results, we hypothesized that the number of cigarettes smoked per day may be associated to the number of streamlines and/or the FA in the genual ROI. As shown in Figure 3B, there was a negative correlation between the number of cigarettes smoked per day and the number of streamlines in the genual ROI (r2 = 0.18, *p* < 0.02). When we studied FA we obtained a similar result but statistically at the trend level (*r*^2^ = 0.1, *p* < 0.08). Consistent with the RSFC data, no correlation was found in the splenium (Figure 3C).

Next, we studied whether specific frontal regions were responsible for the increased interhemispheric connectivity in sated smokers vs. control non-smokers. We studied the interhemispheric connectivity for the following regions: inferior, medial and superior frontal gyri (SFG), striatum (composed by caudate, putamen and globus pallidus), insula, anterior cingulate, and nucleus accumbens. Although almost all regions showed higher connectivity in sated smokers than in controls (Figure 4), only the SFG showed statistical significance (*p*_corrected_ < 0.05). Since most regions showed at least a trend toward more connectivity in sated smokers over non-smokers, it is possible that the effect is widespread.

## Discussion

In the current report, we have shown that cigarette smoking is associated with altered interhemispheric functional connectivity. Specifically, we found that the RSFC between two large seed regions (right and left halves of the brain) is statistically the same between non-smoker controls and deprived smokers. In contrast, sated smokers showed enhanced interhemispheric RSFC. One possible interpretation is that nicotine (and/or other tobacco components) acutely enhances interhemispheric connectivity and that such an effect is short-lived; thus, after 24 h of abstinence the interhemispheric connectivity has returned approximately to basal levels. In addition, we found that the effect of recent smoking on interhemispheric RSFC is more robust in frontal than in caudal regions of the brain. To verify whether the effect of tobacco on interhemispheric RSFC is relevant to smoking behaviors, we correlated the self-reported average number of cigarettes smoked per day with the RSFC between the right and left frontal third of the brain, both in the deprived and the sated condition. We found that there was no correlation between sated frontal interhemispheric RSFC and number of cigarettes per day, but there was a significant positive correlation between average number of cigarettes per day and frontal RSFC during tobacco abstinence. The biological meaning of this observation is not obvious, but it is possible that the correlation was found only in deprived smokers because of a ceiling effect on the much higher connectivity observed under sated conditions. No significant correlations with FTND values were found (not shown) which suggests that the changes in interhemispheric connectivity may be associated with smoking more than with the level of dependence.

To study the possible anatomical substrate for the correlation between cigarettes per day and the frontal interhemispheric RSFC, we performed DTI focusing on two distant regions of the corpus callosum, using manually placed ROIs in the genu and splenium. We found that although the number of streamlines is statistically the same between smokers and controls, the FA of both callosal ROIs (genu and splenium) was markedly diminished in smokers. This is in line with data showing that smokers tend to have lower FA in callosal regions (Lin et al., 2013; Savjani et al., 2014). When we correlated number of cigarettes per day to number or streamlines in the genual ROI we obtained a significant negative correlation. When we used FA we found the same result but it did not reach statistical significance. In close parallel with the RSFC results, no statistical significance was found when the number of streamlines and the FA in the splenium were correlated with cigarettes per day. White matter fibers are the means by which functional connectivity occurs. In the studied sample, we found that decreased streamlines but increased RSFC correlate with increased number of cigarettes per day. An exact parallel between DTI connectivity and RSFC may not be necessarily expected, given that current knowledge about the underpinnings of both DTI measures and RSFC are incomplete. Our data suggests that decreased number of streamlines and increased interhemispheric RSFC are correlated to the same behavioral phenotype. Several explanations are possible for this unexpected finding. First, the interhemispheric RSFC measure is anatomically gross, and thus we are averaging several regions that are functionally very different. It is possible that only some regions are responsible for the observed effect, and that these two observations (increased RSFC and decreased FA) are mainly independent from each other. Second, it has been shown that nicotine has acute effects on white matter properties as measured by DTI (Kochunov et al., 2013). Thus, it is possible that some of the effects we observed on DTI parameters are acute, which should not necessarily be expected to positively correlate with RSFC. Third, there may be compensatory mechanisms that increase functional connectivity (at least within some areas) precisely because white matter tracks are disrupted. Finally, it is possible that the relationship between DTI and RSFC is not as straightforward as expected, and there are examples in the literature where FA and RSFC were observed to move in opposite directions (Qi et al., 2012; Yan et al., 2012). In line with our results, cannabis users have been shown to display decreased callosal FA (Arnone et al., 2008), but increased interhemispheric connectivity (Orr et al., 2013). However, since cannabis is often used together with tobacco, the interpretation of these results may be tricky. Finally, we studied whether some anatomically defined areas were responsible for the increased interhemispheric connectivity in sated smokers. Although the results point to the SFG as the most relevant area, almost all regions showed a trend in the same direction, making it hard to pinpoint the interhemispheric connectivity effect of tobacco to a single defined region. It has been shown that brain size may account for differences in inter- vs. intra hemispheric connectivity (Hänggi et al., 2014). In that regard, we found no differences in total brain size between groups.

Several limitations to this study should be highlighted. First, the effects of age, ethnicity,and sex cannot be reliably studied with our sample size, and we cannot rule out an effect of those or other variables that we did not control. Second, FA measurements in DTI have been shown to be more reliable than streamlines; hence, we tested the 15 possible correlations between FA and number of cigarettes smoked. Even though the correlation was not significant, the trend was similar when compared to streamlines vs. number of cigarettes smoked. While streamlines may not imply distinct fibers, many of the problems associated with analysis of streamlines arise from normalization, a step we carefully avoided (Singh et al., 2010). Finally, for practical reasons we measured several of the subjects twice in the same order (deprived first). This is only a problem for the deprived/sated and control/sated comparisons, and given that most of the data presented is a control/deprivation comparison, there should be no effects of the repeated measure.

Importantly, interhemispheric RSFC is sensitive to repetitive transcranial magnetic stimulation (TMS; Watanabe et al., 2014) and transcranial direct current stimulation (tDCS). Studies on healthy controls have shown that tDCS targeted to the prefrontal cortex can modulate (increase or decrease depending on the parameters used) the risk-taking behavior in gambling tasks (Knoch et al., 2006; Fecteau et al., 2007). Thus, we postulate that frontal interhemispheric RSFC may be a target for TMS or tDCS-based tobacco cessation therapies (Barr et al., 2011; Pedron et al., 2014).

## Conclusion

In conclusion, our data suggest that interhemispheric RSFC, especially in frontal brain regions, may be affected by tobacco smoking and tobacco deprivation.

## Conflict of Interest Statement

The authors declare that the research was conducted in the absence of any commercial or financial relationships that could be construed as a potential conflict of interest.

## References

[B1] Anderson J. S. Druzgal T. J. Froehlich A. DuBray M. B. Lange N. Alexander A. L.. (2011). Decreased interhemispheric functional connectivity in autism. Cereb. Cortex 21, 1134–1146. 10.1093/cercor/bhq190 20943668 PMC3077433

[B2] Arnone D. Barrick T. R. Chengappa S. Mackay C. E. Clark C. A. Abou-Saleh M. T. (2008). Corpus callosum damage in heavy marijuana use: preliminary evidence from diffusion tensor tractography and tract-based spatial statistics. Neuroimage 41, 1067–1074. 10.1016/j.neuroimage.2008.02.064 18424082

[B3] Ashburner J. Friston K. J. (1999). Nonlinear spatial normalization using basis functions. Hum. Brain Mapp. 7, 254–266. 10.1002/(sici)1097-0193(1999)7:4<254::aid-hbm4>3.3.co;2-7 10408769 PMC6873340

[B4] Baldwin P. R. Alanis R. Salas R. (2011). The role of the Habenula in nicotine addiction. J. Addict. Res. Ther. S1:002. 10.4172/2155-6105.S1-002 22493758 PMC3321348

[B5] Barr M. S. Farzan F. Wing V. C. George T. P. Fitzgerald P. B. Daskalakis Z. J. (2011). Repetitive transcranial magnetic stimulation and drug addiction. Int. Rev. Psychiatry 23, 454–466. 10.3109/09540261.2011.618827 22200135

[B6] Benowitz N. L. (2008). Neurobiology of nicotine addiction: implications for smoking cessation treatment. Am. J. Med. 121(4 Suppl. 1), S3–S10. 10.1016/j.amjmed.2008.01.015 18342164

[B7] Benowitz N. L. (2009). Pharmacology of nicotine: addiction, smoking-induced disease and therapeutics. Annu. Rev. Pharmacol. Toxicol. 49, 57–71. 10.1146/annurev.pharmtox.48.113006.094742 18834313 PMC2946180

[B8] Biswal B. B. Mennes M. Zuo X. N. Gohel S. Kelly C. Smith S. M.. (2010). Toward discovery science of human brain function. Proc. Natl. Acad. Sci. U S A 107, 4734–4739. 10.1073/pnas.0911855107 20176931 PMC2842060

[B9] Chen J. L. Schlaug G. (2013). Resting state interhemispheric motor connectivity and white matter integrity correlate with motor impairment in chronic stroke. Front. Neurol. 4:178. 10.3389/fneur.2013.00178 24223571 PMC3819700

[B10] Fecteau S. Knoch D. Fregni F. Sultani N. Boggio P. Pascual-Leone A. (2007). Diminishing risk-taking behavior by modulating activity in the prefrontal cortex: a direct current stimulation study. J. Neurosci. 27, 12500–12505. 10.1523/jneurosci.3283-07.2007 18003828 PMC6673338

[B11] Garvey A. J. Bliss R. E. Hitchcock J. L. Heinold J. W. Rosner B. (1992). Predictors of smoking relapse among self-quitters: a report from the normative aging study. Addict. Behav. 17, 367–377. 10.1016/0306-4603(92)90042-t 1502970

[B12] Gazzaniga M. S. (1967). The human brain is actually two brains, each capable of advanced mental functions. When the cerebrum is divided surgically, it is as if the cranium contained two separate spheres of consciousness. Sci. Am. 217, 24–29. 10.1038/scientificamerican0867-24 4962491

[B13] Guo W. Liu F. Dai Y. Jiang M. Zhang J. Yu L.. (2013a). Decreased interhemispheric resting-state functional connectivity in first-episode, drug-naive major depressive disorder. Prog. Neuropsychopharmacol. Biol. Psychiatry 41, 24–29. 10.1016/j.pnpbp.2012.11.003 23159796

[B14] Guo W. Liu F. Xue Z. Gao K. Liu Z. Xiao C.. (2013b). Decreased interhemispheric coordination in treatment-resistant depression: a resting-state fMRI study. PLoS One 8:e71368. 10.1371/journal.pone.0071368 23936504 PMC3732240

[B15] Hänggi J. Fovenyi L. Liem F. Meyer M. Jäncke L. (2014). The hypothesis of neuronal interconnectivity as a function of brain size-a general organization principle of the human connectome. Front. Hum. Neurosci. 8:915. 10.3389/fnhum.2014.00915 25426059 PMC4227509

[B16] Heatherton T. F. Kozlowski L. T. Frecker R. C. Fagerström K. O. (1991). The Fagerström test for nicotine dependence: a revision of the Fagerström tolerance questionnaire. Br. J. Addict. 86, 1119–1127. 10.1111/j.1360-0443.1991.tb01879.x 1932883

[B17] Hughes J. R. Keely J. Naud S. (2004). Shape of the relapse curve and long-term abstinence among untreated smokers. Addiction 99, 29–38. 10.1111/j.1360-0443.2004.00540.x 14678060

[B18] Janes A. C. Nickerson L. D. Frederick Bde B. Kaufman M. J. (2012). Prefrontal and limbic resting state brain network functional connectivity differs between nicotine-dependent smokers and non-smoking controls. Drug Alcohol Depend. 125, 252–259. 10.1016/j.drugalcdep.2012.02.020 22459914 PMC3389311

[B19] Kelly C. Zuo X. N. Gotimer K. Cox C. L. Lynch L. Brock D.. (2011). Reduced interhemispheric resting state functional connectivity in cocaine addiction. Biol. Psychiatry 69, 684–692. 10.1016/j.biopsych.2010.11.022 21251646 PMC3056937

[B20] Knoch D. Gianotti L. R. Pascual-Leone A. Treyer V. Regard M. Hohmann M.. (2006). Disruption of right prefrontal cortex by low-frequency repetitive transcranial magnetic stimulation induces risk-taking behavior. J. Neurosci. 26, 6469–6472. 10.1523/jneurosci.0804-06.2006 16775134 PMC6674035

[B21] Kochunov P. Du X. Moran L. V. Sampath H. Wijtenburg S. A. Yang Y.. (2013). Acute nicotine administration effects on fractional anisotropy of cerebral white matter and associated attention performance. Front. Pharmacol. 4:117. 10.3389/fphar.2013.00117 24065920 PMC3776159

[B22] Lerman C. Gu H. Loughead J. Ruparel K. Yang Y. Stein E. A. (2014). Large-scale brain network coupling predicts acute nicotine abstinence effects on craving and cognitive function. JAMA Psychiatry 71, 523–530. 10.1001/jamapsychiatry.2013.4091 24622915 PMC4097018

[B23] Li W. Li Q. Zhu J. Qin Y. Zheng Y. Chang H.. (2013). White matter impairment in chronic heroin dependence: a quantitative DTI study. Brain Res. 1531, 58–64. 10.1016/j.brainres.2013.07.036 23895765

[B24] Liao Y. Tang J. Deng Q. Deng Y. Luo T. Wang X.. (2011). Bilateral fronto-parietal integrity in young chronic cigarette smokers: a diffusion tensor imaging study. PLoS One 6:e26460. 10.1371/journal.pone.0026460 22069452 PMC3206030

[B25] Lin F. Wu G. Zhu L. Lei H. (2013). Heavy smokers show abnormal microstructural integrity in the anterior corpus callosum: a diffusion tensor imaging study with tract-based spatial statistics. Drug Alcohol Depend. 129, 82–87. 10.1016/j.drugalcdep.2012.09.013 23062873

[B26] Mansvelder H. D. McGehee D. S. (2002). Cellular and synaptic mechanisms of nicotine addiction. J. Neurobiol. 53, 606–617. 10.1002/neu.10148 12436424

[B27] Moran-Santa Maria M. M. Hartwell K. J. Hanlon C. A. Canterberry M. Lematty T. Owens M.. (2015). Right anterior insula connectivity is important for cue-induced craving in nicotine-dependent smokers. Addict. Biol. 20, 407–414. 10.1111/adb.12124 24529072 PMC4133311

[B28] Narayana P. A. Herrera J. J. Bockhorst K. H. Esparza-Coss E. Xia Y. Steinberg J. L.. (2014). Chronic cocaine administration causes extensive white matter damage in brain: diffusion tensor imaging and immunohistochemistry studies. Psychiatry Res. 221, 220–230. 10.1016/j.pscychresns.2014.01.005 24507117 PMC3943678

[B29] Orr C. Morioka R. Behan B. Datwani S. Doucet M. Ivanovic J.. (2013). Altered resting-state connectivity in adolescent cannabis users. Am. J. Drug Alcohol Abuse 39, 372–381. 10.3109/00952990.2013.848213 24200207

[B30] Pedron S. Monnin J. Haffen E. Sechter D. Van Waes V. (2014). Repeated transcranial direct current stimulation prevents abnormal behaviors associated with abstinence from chronic nicotine consumption. Neuropsychopharmacology 39, 981–988. 10.1038/npp.2013.298 24154668 PMC3924532

[B31] Peto R. Lopez A. D. Boreham J. Thun M. Heath C. Jr. Doll R. (1996). Mortality from smoking worldwide. Br. Med. Bull. 52, 12–21. 10.1093/oxfordjournals.bmb.a011519 8746293

[B32] Pettigrew J. D. Miller S. M. (1998). A ‘sticky’ interhemispheric switch in bipolar disorder? Proc. Biol. Sci. 265, 2141–2148. 10.1098/rspb.1998.0551 9872002 PMC1689515

[B33] Qi R. Xu Q. Zhang L. J. Zhong J. Zheng G. Wu S.. (2012). Structural and functional abnormalities of default mode network in minimal hepatic encephalopathy: a study combining DTI and fMRI. PLoS One 7:e41376. 10.1371/journal.pone.0041376 22911787 PMC3401202

[B34] Savjani R. R. Velasquez K. M. Thompson-Lake D. G. Y. Baldwin P. R. Eagleman D. M. De La Garza R.. (2014). Characterizing white matter changes in cigarette smokers via diffusion tensor imaging. Drug Alcohol Depend. 145, 134–142. 10.1016/j.drugalcdep.2014.10.006 25457737

[B35] Singh M. Jeong J. Hwang D. Sungkarat W. Gruen P. (2010). Novel diffusion tensor imaging methodology to detect and quantify injured regions and affected brain pathways in traumatic brain injury. Magn. Reson. Imaging 28, 22–40. 10.1016/j.mri.2009.05.049 19608369 PMC2789859

[B36] Spencer K. M. Nestor P. G. Niznikiewicz M. A. Salisbury D. F. Shenton M. E. McCarley R. W. (2003). Abnormal neural synchrony in schizophrenia. J. Neurosci. 23, 7407–7411. 12917376 10.1523/JNEUROSCI.23-19-07407.2003 PMC2848257

[B37] Tanabe J. Nyberg E. Martin L. F. Martin J. Cordes D. Kronberg E.. (2011). Nicotine effects on default mode network during resting state. Psychopharmacology (Berl) 216, 287–295. 10.1007/s00213-011-2221-8 21331518 PMC3486925

[B38] Turk D. J. Heatherton T. F. Macrae C. N. Kelley W. M. Gazzaniga M. S. (2003). Out of contact, out of mind: the distributed nature of the self. Ann. N Y Acad. Sci. 1001, 65–78. 10.1196/annals.1279.005 14625356

[B39] Viswanath H. Velasquez K. M. Savjani R. Molfese D. L. Curtis K. Molfese P. J.. (2015). Interhemispheric insular and inferior frontal connectivity are associated with substance abuse in a psychiatric population. Neuropharmacology 92C, 63–68. 10.1016/j.neuropharm.2014.12.030 25592214

[B40] Volpato C. Piccione F. Cavinato M. Duzzi D. Schiff S. Foscolo L.. (2013). Modulation of affective symptoms and resting state activity by brain stimulation in a treatment-resistant case of obsessive-compulsive disorder. Neurocase 19, 360–370. 10.1080/13554794.2012.667131 22554168

[B41] Wang L. Li K. Zhang Q. E. Zeng Y. W. Jin Z. Dai W. J.. (2013). Interhemispheric functional connectivity and its relationships with clinical characteristics in major depressive disorder: a resting state fMRI study. PLoS One 8:e60191. 10.1371/journal.pone.0060191 23555920 PMC3612036

[B42] Watanabe T. Hanajima R. Shirota Y. Ohminami S. Tsutsumi R. Terao Y.. (2014). Bidirectional effects on interhemispheric resting-state functional connectivity induced by excitatory and inhibitory repetitive transcranial magnetic stimulation. Hum. Brain Mapp. 35, 1896–1905. 10.1002/hbm.22300 23897535 PMC6869044

[B43] Whitfield-Gabrieli S. Nieto-Castanon A. (2012). Conn: a functional connectivity toolbox for correlated and anticorrelated brain networks. Brain Connect. 2, 125–141. 10.1089/brain.2012.0073 22642651

[B44] Wylie K. P. Rojas D. C. Tanabe J. Martin L. F. Tregellas J. R. (2012). Nicotine increases brain functional network efficiency. Neuroimage 63, 73–80. 10.1016/j.neuroimage.2012.06.079 22796985 PMC3429645

[B45] Yan H. Tian L. Yan J. Sun W. Liu Q. Zhang Y. B.. (2012). Functional and anatomical connectivity abnormalities in cognitive division of anterior cingulate cortex in schizophrenia. PLoS One 7:e45659. 10.1371/journal.pone.0045659 23049832 PMC3458074

[B46] Zorlu N. Karavul Ucman T. Gelal F. Colak Kalayci C. Polat S. Saricicek A.. (2014). Abnormal white matter integrity in long-term abstinent alcohol dependent patients. Psychiatry Res. 224, 42–48. 10.1016/j.pscychresns.2014.07.006 25104315

